# Fabrication of Multi-Layered Lidocaine and Epinephrine-Eluting PLGA/Collagen Nanofibers: In Vitro and In Vivo Study

**DOI:** 10.3390/polym9090416

**Published:** 2017-09-05

**Authors:** Fu-Ying Lee, Demei Lee, Tzu-Chia Lee, Jan-Kan Chen, Ren-Chin Wu, Kuan-Chieh Liu, Shih-Jung Liu

**Affiliations:** 1 Department of Periodontics, Division of Dentistry, Chang Gung Memorial Hospital, Tao-Yuan 33305, Taiwan; fuying20@hotmail.com; 2 Department of Mechanical Engineering, Chang Gung University, Tao-Yuan 33302, Taiwan; dmlee@mail.cgu.edu.tw (D.L.); joy820629@gmail.com (T.-C.L.); harobinca@hotmail.com (K.-C.L.); 3 Department of Physiology and Pharmacology, Chang Gung University, Tao-Yuan 33302, Taiwan; jkc508@mail.cgu.edu.tw; 4 Department of Pathology, Chang Gung Memorial Hospital, Tao-Yuan 33305, Taiwan; renchin.wu@gmail.com; 5 Department of Orthopedic Surgery, Chang Gung Memorial Hospital, Tao-Yuan 33305, Taiwan

**Keywords:** biodegradable nanofibers, PLGA, collagen, epinephrine, lidocaine

## Abstract

This study developed multi-layered lidocaine- and epinephrine-eluting biodegradable poly[(d,l)-lactide-*co*-glyco lide] (PLGA)/collagen nanofibers. An electrospinning technique was employed to fabricate the multi-layer biodegradable drug-eluting nanofibers. After fabrication, the nanofibrous membranes were characterized. The drug release characteristics were also investigated. In addition, the in vivo efficacy of nanofibers for pain relief and hemostasis in palatal oral wounds of rabbits were evaluated. Histological examinations were also completed. The experimental results suggested that all nanofibers exhibited good biocompatibility and eluted effective levels of lidocaine and epinephrine at the initial stages of wound recovery.

## 1. Introduction

A free gingival graft (FGG) is a dental procedure that involves harvesting soft tissue from a distant site in the mouth and grafting it over a localized recession defect. FGG was first introduced in 1963 and found the clinical applications to treat mucogingival lesions one year later [1,2,3]. After the procedure, tissues at the excisional wound are replaced by fibrin and inflammatory cells. It is a surgical procedure widely employed to enhance the keratinized tissue encompassing a tooth and/or a dental implant. The technique has also been extensively employed to treat various periodontal mucogingival lesions including insufficient or lack of attached gingiva, the presence of high frenum attachments, shallow fornix, and denuded roots following gingival recession. However, the donor tissue is usually taken from the palate. The postoperative response at a palate wound site is generally uneventful. In addition, excessive bleeding and fierce post-surgery pain have been addressed after the FGG procedure [4,5,6,7].

For decades, science has recognized that wound dressings are extremely important components of wound care and are necessary for optimal wound healing. Open palatal wound recovery consists of a dynamic and intense process, which involves blood clot deposition, inflammation, and angiogenesis to constitute a vascularized granulation tissue. After that, an approximation of epithelium and tissue remodeling is completed to create a mature recovery process in four weeks [8]. Wound dressings perform three basic functions: protect wounds, help prevent infection, and maintain optimal moisture, consequently accelerating the healing process.

Lidocaine is a drug widely used to numb tissue in a specific area and may be applied directly to the skin for numbing [9]. Epinephrine, on the other hand, is primarily a medication used for a number of conditions including anaphylaxis, cardiac arrest, and superficial bleeding [10]. When lidocaine is mixed with a small amount of epinephrine, it allows larger doses to be used for numbing and increases the length of time it is effective [11]. Type I collagen is the most abundant collagen in the body that constitutes collagen fibers [12,13]. It is also one component of skin tissue that benefits all stages of the wound healing process. McGuire and Scheyer [14] employed a xenogeneic collagen matrix as a substitute to FGG for the augmentation of oral soft tissues. Their results showed that the collagen matrix may act as a suitable substitute for FGG in vestibuloplasty procedures designed to improve keratinized tissue around teeth.

This study developed biodegradable lidocaine, epinephrine, and collagen loaded nanofibrous membranes that offered a sustainable elution of hemostatic and analgesic drugs at the oral wound sites to decrease postoperative pain and accelerate palatal donor site wound healing. An electrospinning technique was employed to fabricate the multi-layer biodegradable drug-eluting nanofibrous membranes. The technique has been widely employed to fabricate nanofibers of various materials as well as nanofibrous membranes encapsulating living cells [15]. After fabrication, the nanofibrous membranes were characterized. The drug release characteristics were investigated. The efficacy of the drug-loaded membranes was assessed on a rabbit model. Histological analysis was completed as well.

## 2. Materials and Methods

### 2.1. In Vitro Release of Epinephrine and Lidocaine

#### 2.1.1. Manufacture of Polycaprolactone Stent and Drugs Loaded PLGA Nanofibers

The polymeric materials included poly(ε-caprolactone) (PCL) (Sigma-Aldrich, St. Louis, MO, USA) and poly[(d,l)-lactide-*co*-glyco lide] (PLGA) polymer (lactide/glycolide: 50/50) (Sigma-Aldrich, St. Louis, MO, USA). Epinephrine and lidocaine hydrochloride were included, while type I collagen was from calf skin (Sigma-Aldrich, St. Louis, MO, USA).

A PCL stent with a dimension of 9 mm × 12 mm and an open window of 5 mm × 5 mm was sliced from a 0.15 mm thick film that is solvent-casted. Membranes with three various PLGA/drug combinations, namely 6:1, 3:1 and 2:1, respectively, were produced. To manufacture the multi-layered membranes with 6:1 polymer-to-drug ratio, PLGA/lidocaine (300 mg:50 mg), PLGA/epinephrine (300 mg:50 mg), and PLGA/collagen (300 mg:50 mg) were primarily dissolved in 1 mL of hexafluoroisopropanol (HFIP) (Sigma-Aldrich), respectively. The PLGA/lidocaine solution was spun and collected by the collection plate in a nonwoven form. The procedure was duplicated to manufacture subsequently the PLGA/epinephrine and PLGA/collagen nanofibers. All procedures were performed at room temperature. On the other hand, the preparation of nanofibrous membranes with 3:1 and 2:1 polymer-to-drug ratios followed the same electrospinning procedure, except that the polymer/drug used were 300 mg/100 mg and 300 mg/150 mg, respectively.

#### 2.1.2. Characterization of Electrospun Nanofibrous Membranes

A field emission scanning electron microscope (FE-SEM) (JSM-7500F, Joel, Japan) was employed to quantify the electrospun nanofibers. The diameter compositions of nanofibers were acquired by characterizing the microphotos of 50 arbitrarily picked fibers for each specimen.

The spectra of electrospun nanofibrous membranes were characterized using a Fourier Transform Infrared (FTIR) spectrometry. FTIR analysis was completed on a Nicolet iS5 spectrometer (Thermo Fisher Scientific, Waltham, MA, USA) at a resolution of 4 cm^−1^ and 32 scans. Nanofibrous membranes were pressed as KBr discs, and spectra were analyzed over the 400–4000 cm^−1^ range.

The tensile characteristics of electrospun nanofibrous membranes were quantified on a Lloyd tensiometer (AMETEK, Berwyn, PA, USA).

The water contact angles of the nanofibers were assessed by a contact angle measuring apparatus (First Ten Angstroms, Portsmouth, VA, USA) (N = 5).

#### 2.1.3. Drug Concentration Assessment

The elution behavior of lidocaine and epinephrine from the drug-loaded nanofibers with various PLGA/drug combinations, namely 6:1, 3:1 and 2:1, respectively, was evaluated. Specimens, with an approximate size of 2.0 mm × 3.0 mm and weight of 1 g, were sliced from the electrospun membrane and stored in glass tubes. Each tube contained one specimen along with 1 mL of buffered solution (0.15 mol/L, pH 7.4). All test tubes were maintained at 37 °C for 24 h. Then the eluent was collected for analysis, and fresh buffered solution (1 mL) was added. The procedure was repeated every 24 h until the specimen was fully dissolved. The experiment was triplicated (N = 3).

The lidocaine levels in the eluents were quantified using a high performance liquid chromatography (HPLC) assay. The HPLC analyses were carried out on a Hitachi L-2200R system, using an ATLANTIS dC18, 4.6 cm × 150 mm column (Waters Corp., Milford, MA, USA). Ammonium formate and methanol (Sigma-Aldrich; 20/80 (*v*/*v*)) were adopted as the mobile phase. The absorbency of monitoring was set at a wavelength of 210 nm, while the flow rate was maintained at 1.0 mL/min. To characterize epinephrine, a Discovery BIO Wide C18-5, 25 cm × 4.6 mm column was used. The mobile phase contained water, methanol, and acetic acid (Sigma-Aldrich) in a volume ratio of 85:10:5. The pH value of the mobile phase was adjusted by adding ammonium acetate (Sigma-Aldrich) to reach 3.1. The absorbency was maintained at 280 nm, while the flow rate was set at 1.0 mL/min. All specimens were assayed in triplicate (N = 3).

### 2.2. Cytotoxicity of Composite Nanofibers

Cytotoxicity of fabricate nanofibers was assessed by MTT assay (Roche, Berlin, Germany). Electrospun nanofibers were cut out with punch and placed onto 24-well culture plates. Human fibroblasts were seeded (5 × 10^3^ cells/well) in DMEM at 37 °C under 5% CO_2_/95% air conditions until cell confluence. Cell viability was observed on Days 1, 2, 3, and 7 by MTT assays and evaluated using an ELISA reader. The number of cells was also calculated under an optical microscope (Olympus IMT-2, Tokyo, Japan).

### 2.3. In Vivo Study of Animal

#### 2.3.1. Animal-Related Procedure

Twelve New Zealand white rabbits were enrolled for the in vivo study. The average weight of the animals was 3.3 kg. All animal-related processes obtained the approval from the institution. The animals were taken care of following the guidelines of the Department of Health and Welfare, Taiwan.

The rabbit was sedated with 2% xylazine-HCl (5 mg/kg body weight) and Ketamine-HCl (Ketasol, 30 mg/kg body weight). The operative area was cleaned and sterilized. All surgical procedures were carried out using a #15 blade after the local injection of 0.5 mL 2% Xylestesin-A containing epinephrine at a concentration of 1:100,000. A rectangular shape incision of 6 mm × 8 mm (width × length) and 1–1.5 mm in thickness was created on the palate of rabbits. After eliminating the graft, wet gauze was applied onto the donor site for 60 s with moderate finger pressure.

**Figure 1:**
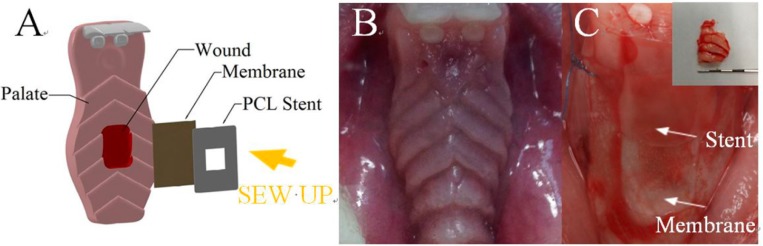
(**A**) Schematic of the deployment of biodegradable stent and nanofibrous membrane on the donor site; and (**B**) beforel and (**C**) after the deployment of stent and membrane.

The 12 rabbits were stochastically separated into two groups: In the control group, the excision wound was covered by a PCL stent without membrane and sutured with 6-0 polypropylene suture at the corners of the stent (Figure 1). In the test group, the donor site was covered by a PCL stent with multi-layered lidocaine/epinephrine/collagen loaded biodegradable nanofibrous membrane. For simplicity reason, the membrane with the PLGA/drug combination of 3:1 was employed. After that, the stent was sutured with 6-0 polypropylene suture at the corners.

In the test group, the tissue fluid at the palatal excision wound was extracted using #30 standardized sterile paper point (DiaDent, Korea) on Days 1, 2, 4, 7, 10 and 14. Sampling was performed in situ for 30 s during each procedure. Immediately after collection, the paper point was eluted with 0.1 mL phosphate buffer solution and then stored at −20 °C until it was analyzed. The HPLC analysis was employed to characterize the drug concentrations at each time period, using the same procedure as the in vitro study.

#### 2.3.2. Post-Surgery Evaluation

The daily changes of body weight, and food/water intakes were monitored post-surgery for 14 days. In addition, the hemostasis analysis was completed following the work of Saroff et al. [16].

#### 2.3.3. Wound Healing and Histological Analysis

On Days 1, 3, 7 and 14 after operation, photos of the wound for each rabbit were taken. To evaluate and compare the tissue responses at a microscopically level, standard biopsy was done at the end of Days 1, 3, 7 and 14. The specimens were harvested and fixed in 10% formalin and embedded in paraffin, cut into 4 μm frontal section. The epithelial and connective tissue characteristics were examined by employing the Hematoxylin and Eosin (H&E) staining. The interpretation was completed at ×10 and ×20 magnification by an independent examiner.

### 2.4. Statistical Analysis

The differences between groups were assessed using a least significance difference test, by employing SPSS software (SPSS Inc., Armonk, NY, USA).

## 3. Results

### 3.1. In Vitro Characterization of Electrospun Drug-Loaded Nanofibrous Membranes

**Figure 2:**
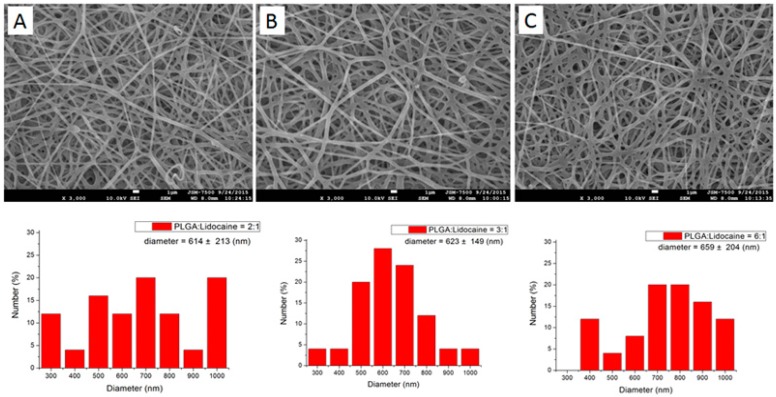
Scanning electron microscopy (SEM) photos and fiber diameter distribution of electrospun drugs loaded nanofibers. PLGA:Lidocaine (**A**) 2:1 (**B**) 3:1 (**C**) 6:1.

Figure 2 shows the SEM microphotos of the drugs loaded nanofibers and the diameter compositions of each fiber. The calculated diameters were 614 ± 213 nm, 623 ± 149 nm, and 659 ± 204 nm, respectively, for the 2:1, 3:1 and 6:1 lidocaine/epinephrine loaded PLGA/collagen nanofibers. The fiber diameter increased slightly with the percentage of polymers in the membranes. When subjected to an external force exerted by the electric field, the polymeric solution that has a higher polymer percentage showed a higher strength and was less extended. Electrospun nanofibers thus exhibited greater fiber diameters.

**Figure 3:**
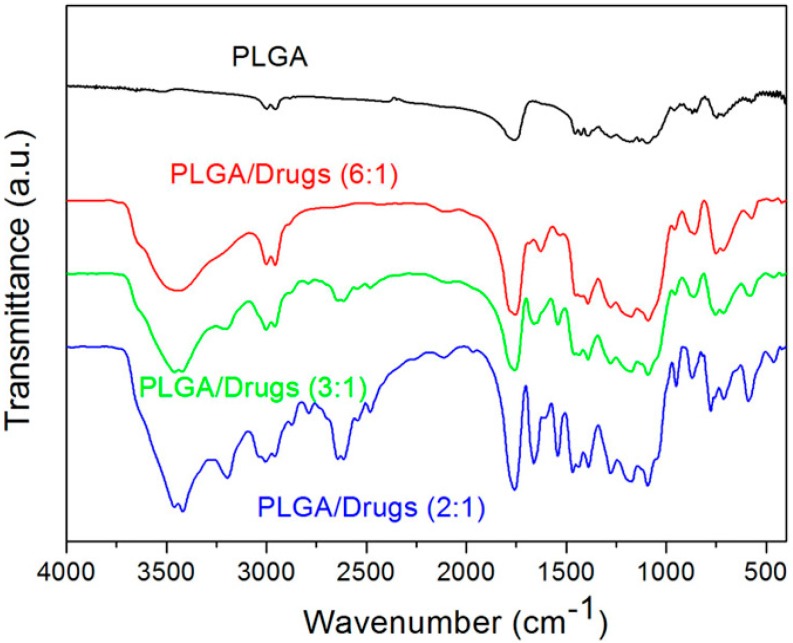
Fourier Transform Infrared (FTIR) spectra of electrospun nanofibrous membranes. The results of the FTIR spectra demonstrated the incorporation of drugs in the poly[(d,l)-lactide-*co*-glyco lide] (PLGA) matrix.

Figure 3 displays the calculated spectra of non-drug loaded PLGA nanofibers and drug incorporated PLGA nanofibers. The absorption peaks of epinephrine and lidocaine were identified in electrospun PLGA nanofibers. The vibration peak at 3400 cm^−1^ can be attributed to the N-H bonds of lidocaine and epinephrine, while the vibration at 3250 cm^−1^ of the O–H bond was improved with the presence of the drugs. In addition, the absorbance at the region of 3200 cm^−1^ could be resulted from the benzene of lidocaine [17] and epinephrine [18]. The results of the FTIR spectra demonstrated the incorporation of drugs in the PLGA matrix.

Measured water contact angles of pure PLGA, and 6:1, 3:1 and 2:1 PLGA:drug ratio loaded nanofibrous membranes were 127.4°, 118.5°, 87.6° and 54.6°, respectively. The addition of lidocaine and epinephrine increased the hydrophilicity of the PLGA nanofibers. Furthermore, the water contact angle (hydrophilicity) increased with the content of drugs in electrospun nanofibers.

The tensile test results demonstrated that the maximum strength (elongation at break) of the pure PLGA, and 6:1, 3:1 and 2:1 PLGA:drug ratio loaded nanofibers were 4.53 MPa (229.7%), 2.85 MPa (51.2%), 1.87 MPa (37.0%) and 1.40 MPa (26.1%), respectively. Obviously, the mechanical strengths decreased with the content of incorporated drugs.

**Figure 4:**
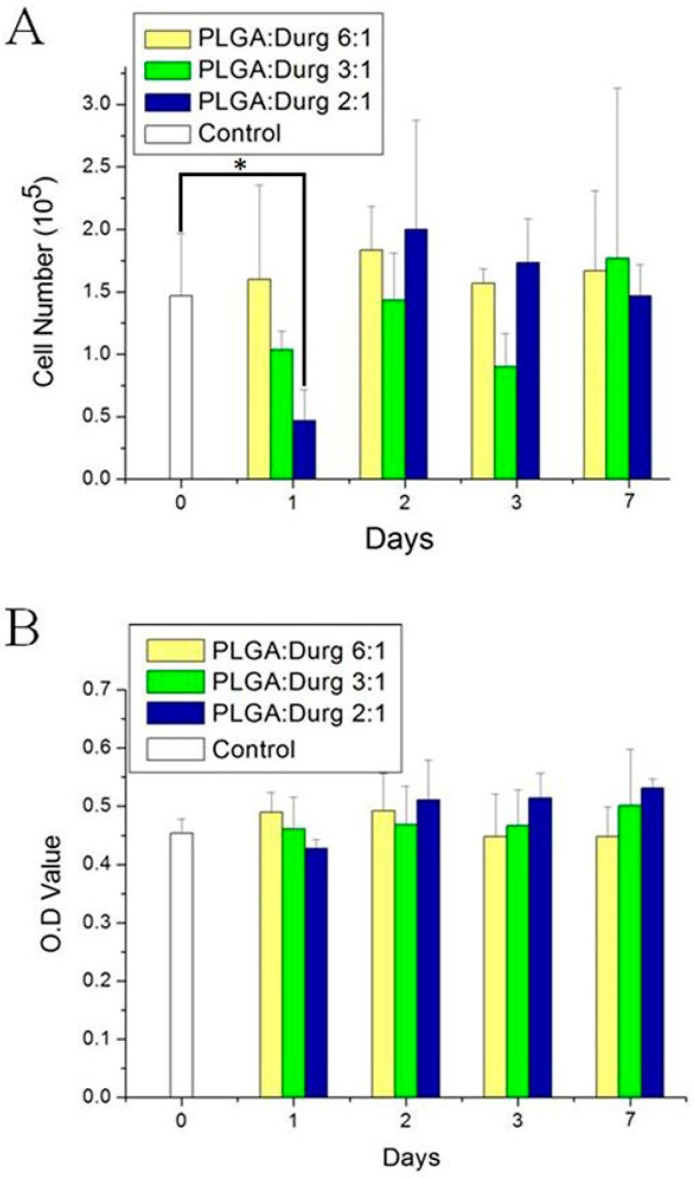
Toxicity of electrospun nanofibrous composite membranes (*p* < 0.05). The fabricated nanofibers show no signs of cytotoxicity. (**A**) cell number; (**B**) optical density (OD) value.

Cytotoxicity experiments from MTT assays of the composite nanofibers were examined. The measured result in Figure 4 suggested that the fabricated nanofibers do not show any signs of cytotoxicity.

### 3.2. In Vitro Elution Characteristics of Epinephrine and Lidocaine

**Figure 5:**
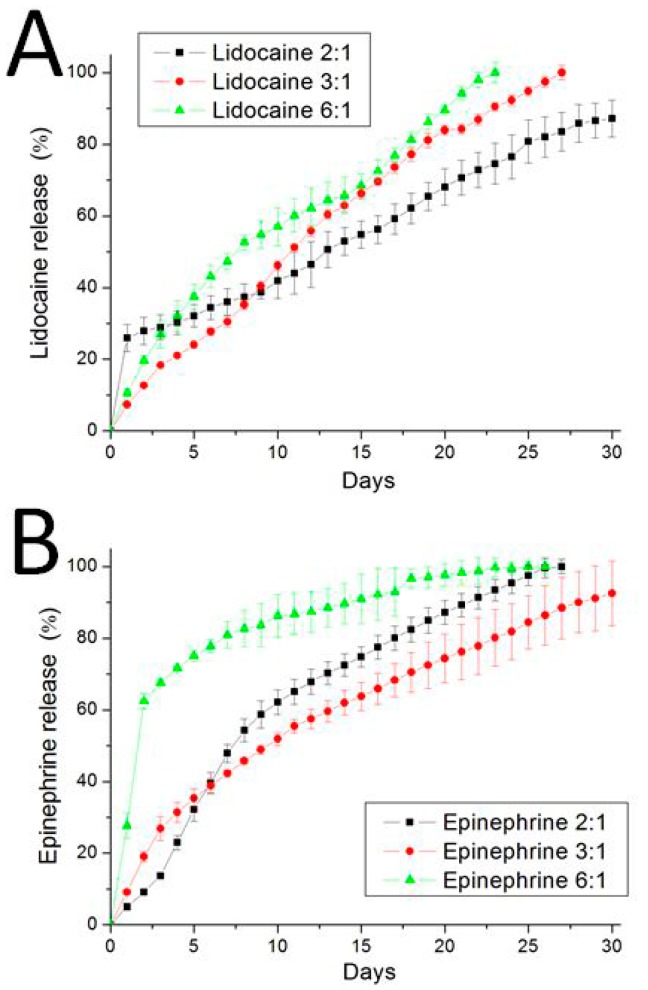
Release curves of: (**A**) lidocaine and (**B**) epinephrine from the nanofibrous membranes. All biodegradable nanofibers released effective concentrations of epinephrine and lidocaine for over three weeks.

Figure 5A shows the elution behaviors of lidocaine, while Figure 5B displays the release patterns of epinephrine. The 6:1 polymer-to-drug ratio membrane showed a biphasic release profile, consisting of a burst elution during the first two days and a steady and gradually diminishing drug release. The elution pattern was comparable for the 3:1 and 2:1 polymer-to-drug ratios nanofibrous membranes, with a steadily decreasing release, indicating that the lidocaine and epinephrine were evenly encapsulated and distributed in the electrospun nanofibrous membranes. In addition, all biodegradable nanofibers released effective concentrations of epinephrine and lidocaine for over three weeks.

### 3.3. In Vivo Elution of Lidocaine and Epinephrine

**Figure 6:**
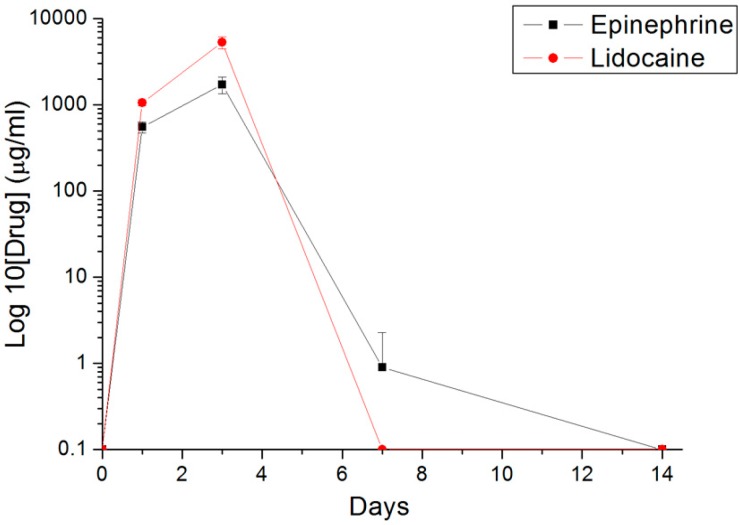
In vivo release of lidocaine and epinephrine from the nanofibrous membranes. Release peaks were observed at Days 1 and 3, after which the drug concentration dropped significantly at Day 7.

Figure 6 shows the in vivo elution profiles of lidocaine and epinephrine from the nanofibrous membranes. Release peaks were observed at Days 1 and 3, after which the drug concentration dropped significantly at Day 7 because both the stent and the membrane were not found at the wound sites by Day 7, possibly removed by the rabbit’s tongue and swallowed by the rabbit. Measured drug level thus decreased accordingly.

### 3.4. Efficacy of Released Drugs

#### 3.4.1. Hemostasis Efficacy

The hemostasis efficacy of the drugs loaded nanofibrous membranes was evaluated [15]. For the initial hemostasis (1 min after the surgery), 67% (4/6) in the test showed hemostasis outcome, but only 17% (1/6) of wounds displayed hemostasis. Furthermore, while 100% (6/6) of the wounds in the test group exhibited effective hemostasis at 10 min, only 83% (5/6) of the wounds in the control showed hemostasis. The results here demonstrated the hemostasis capability of the drug-loaded collagen nanofibers.

#### 3.4.2. Weight Changes

The preoperative body weights in the control group and the test group were comparable: 2.97 ± 0.43 kg in the control group A and 3.04 ± 0.68 kg in the test group B. No significant difference was found in the mean body weights of the rabbits between the control group and the test group by the end of the study.

#### 3.4.3. Food Intake and Water Consumption

The food intake and water consumption were recorded. On Day 1, food intake by rabbits in the control group decreased significantly when compared to those in the test group. The amount of food intake increased gradually for all rabbits in both groups. On Day 14, rabbits of both groups regained their normal food intake (approximately 135 g). Throughout the study period, the rabbits in the test group exhibited greater food intake than did the rabbits in the control group. However, the difference did not reach significant level.

On the other hand, all rabbits reduced their water consumption by 50% at one day post-operation, after which the water consumption increased gradually. On Day 14, all rabbits regained their normal amount of water consumption (approximately 1100 mL). Again, the rabbits in the test group (composite membrane group) showed greater amount of water consumption than did those in the control group, although the difference is not statistically significant.

#### 3.4.4. Wound Healing and Histological Analysis

**Figure 7:**
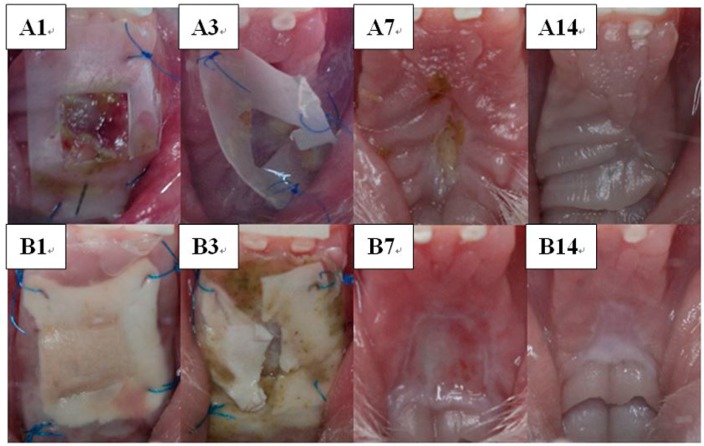
Wound healing in (**A**) control group and (**B**) test (nanofibrous composite membrane) group on Days 1, 3, 7 and 14 post-surgery.

Figure 7 shows the upper gingiva of the rabbits from each group on Days 1, 3, 7 and 14 after surgery. On Day 1, stents of both groups were in situ. The biodegradable composite membranes fully covered the palatal wound. From the window of the control site stent, formation of the granulation tissue could be observed. On Day 3, stents in the control group were sloughing, while the multiple layered nanofibrous membrane was broken at the wound site. Food debris was also found. All stents in both groups were completely disintegrated by Day 7. The gross wound appearance in the control group exhibited necrotic tissue along with new epithelialized gingiva tissues. In contrast, the wound dressed by the multiple layered nanofibrous membrane showed clear wound surface. On Day 14, the wounds in rabbits of both groups were fully recovered.

**Figure 8:**
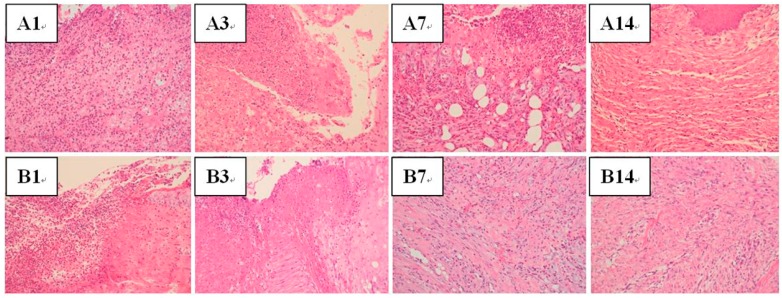
Histological images of (**A**) control group and (**B**) nanofibrous composite membrane group on Days 1, 3, 7 and 14. Polymorphonuclear neutrophils (PMN) infiltration was moderate in the drug-eluting membrane treated wounds, while the wounds in the control group showed heavy PMN infiltration on Day 7.

Figure 8 shows the histological analysis result. On Days 1 and 3, necrosis with heavy Polymorphonuclear neutrophils (PMN) infiltrations were observed in both groups. Despite the fact that active fibrosis had been found in both groups, PMN infiltration was moderate in the drug-eluting membrane treated wounds, while the wounds in the control group showed heavy PMN infiltration on Day 7. On Day 14, fibrosis with few inflammatory cells was found on both groups and no significant difference was found between the groups.

## 4. Discussion

Postoperative pain and bleeding are the most common complications following soft tissue grafting procedures. Pain control is important during the early phase in soft tissue surgery. In the early operative period, free gingival graft accounts for the greater incidence of donor site pain. The pain results in stress and impairs the neutrophil function. Furthermore, the stress affects the immune system and down regulates IL-1 gene expression and impairs wound healing [19]. After the procedures, tissues at excisional wound are substituted by fibrin and inflammatory cells. The palatal excision wound with large epithelial deficiencies cannot cure by initial closure of the wound edges. A secondary recovery with epithelial cell migration from the surrounding to the central region of the defect is usually required to heal the wound [20,21]. Ward was the first to advocate for the adoption of periodontal dressing after gingival surgery so as to decrease pain and infection at the wound site [22]. Various procedures for protecting and covering the donor site, including intraoral bandage or oral adhesive, interproximal wire ligation, surgical dressing by mattress sutures, and modified Hawley retainer, have also been proposed [23]. To facilitate the recovery process and to decrease the occurrence of complications, chemotherapeutic agents and hemostatic agents, ozonides and low-intensity pulse ultrasound, and low-energy laser irradiation were introduced [8,24,25,26].

This study developed multi-layered nanofibrous lidocaine/epinephrine-eluting PLGA membranes that provide good conformity to underlying gingiva, reduce postoperative pain and hemorrhage, and protect the clot from the forces applied during chewing. The drug release kinetics for the biodegradable drug-eluting nanofibrous membranes comprise of two different phases: an initial burst and a degradation-dominated phase. During electrospinning, most drugs are encapsulated and distributed in the nanofibers. Nevertheless, some drug compounds may be located on the nanofiber surface, thus resulting in the initial burst. After that, the drug-elution behavior is mainly decided by polymer degradation. The drugs are released as the polymers degrade with time [27]. The nanofibers thus exhibited a steady and diminishing release of epinephrine and lidocaine. The biodegradable multi-layered drug-eluting PLGA/collagen nanofibrous membranes could release high levels of epinephrine and lidocaine for over four weeks. This would provide advantages for pain and bleeding control and tissue regeneration.

Malmquist et al. [28] studied the HemCone dental dressing (HDD) and showed that it is a clinically effective hemostatic device that significantly reduces bleeding time after oral surgeries. HDD is fabricated from freeze-dried chitosan derived from shrimp shell chitin. Several authors demonstrated that HDD exhibits cell adhesion properties and releases growth factors from human palates stimulated by chitosan [29,30]. Despite the hemostatic capability, small amounts of unreacted residual acetic acid in wounds covered by the HDDs can cause temporary pain in the first two days after surgery [28]. This study employed PLGA as the delivery vehicle of the drugs. PLGA is a biocompatible and biodegradable polymeric material that shows a wide range of degradation times as well as adjustable mechanical properties. It has been widely investigated as delivery carriers for drugs, proteins and various other macromolecules [31,32]. 

Recent studies used platelet-rich fibrin (PRF) for promoting palatal donor wound healing. PRF is a fibrin matrix incorporating and releasing platelet cytokines, growth factors, and cells. The fibrin matrix is the natural guide of initial angiogenesis modulated by the binding to various growth factors. Fibrin plays an important role in modulating neutrophil activity and inducing epithelial cell migration to enhance the process of wound healing [33]. Kulkarni et al. [34] reported that PRF used for palatal wounds promotes complete wound closure at one week, reduces inflammation reaction at the periphery of the healing wound, and displays good control of bleeding at the time of surgery [32]. Although PRF has a positive effect on wound healing, patients may suffer from venous blood donation with another skin wound. In addition, as the preparation is strictly autologous, the amount of PRF obtained is limited.

Collagen is a natural substrate of extracellular matrix. The collagen dressing has the ability to realize hemostasis, chemotactic to fibroblasts and platelets, and induce the proliferation and differentiation of mesenchymal cells. Shanmugam et al. [35] demonstrated that collagen-based dressing offers significantly greater advantages over the traditional non-eugenol dressing in the healing of palatal wounds. As a scaffold, the nanofibrous membranes should be able to enhance cell proliferation and physiological function and retain normal states of cell growth. In this study, the electrospun multi-layered nanofibrous membranes show no signs of cytotoxicity. The possible effect of released acid from the hydrolysis of PLGA nanofibers on cell proliferation was also negligible. Furthermore, the animals dressed with the drug-eluting nanofibers show greater food intake and water consumption than did those without (the control). The measured in vivo release curve (Figure 6) showed that the multi-layered nanofibers release high concentrations of epinephrine and lidocaine during the first three days, which helps relieve pain [11]. This suggests that the fabricated nanofibers may provide effective analgesic effects at the wound site of free tissue grafts and promote wound healings.

It has been proposed that human skin fibroblasts exhibited higher growth on nanofibrous membranes possessing fiber diameters in the range of 350–1100 nm [36]. The measured diameters were 614 ± 213 nm, 623 ± 149 nm and 659 ± 204 nm, respectively, for the 2:1, 3:1 and 6:1 lidocaine/epinephrine loaded PLGA nanofibrous membranes. The fabricated membranes thus show positive cell proliferations in normal human fibroblasts. Despite the fact that lidocaine and epinephrine released from the nanofibers may delay cell growth and proliferation on Day 1, the negative impact of released drugs was found to be negligible starting from Day 2 and forward (Figure 4). On the other hand, the histological analysis images show no inflammation of the tissues. This further supports that the PLGA/drugs/collagen nanofibers can be an appropriate scaffold for tissue regeneration of free grafts.

## 5. Conclusions

This study developed multi-layered lidocaine- and epinephrine-eluting PLGA/collagen nanofibers and evaluated their efficacy for pain relief and hemostasis in palatal oral wounds of rabbits. All nanofibers exhibited good biocompatibility and eluted effective levels of lidocaine and epinephrine at the early stages of wound healing. Rabbits in the test group showed faster hemostasis as well as recovery of food and water intake postoperatively, compared with those in the control group. The experimental results demonstrate that the multi-layered biodegradable nanofibrous membranes offered adequate efficacy of hemostasis and sustainable pain relief for the initial healing of palatal oral wounds.
